# Immunomodulatory Activity of Tyrosine Kinase Inhibitors to Elicit Cytotoxicity Against Cancer and Viral Infection

**DOI:** 10.3389/fphar.2019.01232

**Published:** 2019-10-18

**Authors:** Núria Climent, Montserrat Plana

**Affiliations:** AIDS Research Group, Institut d’Investigacions Biomèdiques August Pi i Sunyer (IDIBAPS), HIV Vaccine Development in Catalonia (HIVACAT), Hospital Clínic de Barcelona, Faculty of Medicine, University of Barcelona, Barcelona, Spain

**Keywords:** tyrosine kinase inhibitors, chronic myeloid leukemia, dasatinib, T cells, adaptive natural killer cells, cytomegalovirus, human immunodeficiency virus, SAMHD1

## Abstract

Tyrosine kinase inhibitors (TKIs) of aberrant tyrosine kinase (TK) activity have been widely used to treat chronic myeloid leukemia (CML) for decades in clinic. An area of growing interest is the reported ability of TKIs to induce immunomodulatory effects with anti-tumor and anti-viral activity, which appears to be mediated by directly or indirectly acting on immune cells. In selected cases of patients with CML, TKI treatment may be interrupted and a non-drug remission may be observed. In these patients, an immune mechanism of increased anti-tumor cytotoxic activity induced by chronic administration of TKIs has been suggested. TKIs increase some populations of natural killer (NK), NK-LGL, and T-LGLs cells especially in dasatinib treated CML patients infected with cytomegalovirus (CMV). In addition, dasatinib increases responses against CMV and is able to inhibit HIV replication *in vitro*. Recent studies suggest that subclinical reactivation of CMV could drive expansion of specific subsets of NK- and T-cells with both anti-tumoral and anti-viral function. Therefore, the underlying mechanisms implicated in the expansion of this increased anti-tumor and anti-viral cytotoxic activity induced by TKIs could be a new therapeutic approach to take into account against cancer and viral infections such as HIV-1 infection. The present review will briefly summarize the immunomodulatory effects of TKIs on T cells, NKs, and B cells. Therapeutic implications for modulating immunity against cancer and viral infections and critical open questions are also discussed.

## Tyrosine Kinase Inhibitors in Chronic Myeloid Leukemia

Chronic myeloid leukemia (CML) is a hematopoietic progenitor cell neoplasm characterized by the uncontrolled growth of myeloid cells in the bone marrow and their accumulation in peripheral blood (D’Antonio, 2007). It is caused by a translocation between chromosomes 9 and 22 that generates an aberrant chromosome 22 called Philadelphia (Ph). At the molecular level, this translocation generates the BCR-ABL oncogene (Andreieva et al., 2016), which encodes a constitutively activated tyrosine kinase (TK), which is the cause of Ph-positive (Ph^+^) CML and Ph^+^ acute lymphoblastic leukemia (ALL) (Quintás-Cardama et al., 2009). The treatment of CML is based on a series of small molecules inhibiting the TK of BCR-ABL, including dasatinib, imatinib, nilotinib, bosutinib, and ponatinib (Jabbour, 2016). Imatinib was the first drug in this family and revolutionized the treatment of CML by achieving high remission rates and improved survival to normal patient ranges (Thompson et al., 2015). Nilotinib, dasatinib, bosutinib, and ponatinib are characterized by a much higher potency than imatinib against BCR-ABL (>20-300) (Simoneau, 2013).

## Ability of Tyrosine Kinase Inhibitors to Induce Immunomodulatory Effects

Due to their different mechanism of action, tyrosine kinase inhibitors (TKIs) cause different immune effects on T-cells, natural killer (NK) cells, and B-cells (Steegmann et al., 2012). Imatinib causes lymphopenia and decreases immunoglobulin levels (Cwynarski et al., 2004), while nilotinib inhibits CD8^+^ T cell function (Chen et al., 2008b). Dasatinib, one of the most potent TKIs, interferes with the activity of several kinases of the Src family that are important for immune response, such as Lck and Fyn in T-lymphocyte signaling, and Lyn, Syk, and Btk in B-lymphocyte signaling (Giansanti et al., 2014). Dasatinib also inhibits proliferation and activation of T lymphocytes and suppresses cytotoxic activity of NK cells (Damele et al., 2018; Marinelli Busilacchi et al., 2018). Imatinib, nilotinib, or dasatinib treatments are correlated with decreased B-lymphocyte functions in CML patients (de Lavallade et al., 2013; Rajala et al., 2017). Leukemia-associated antigen (LAA)-specific CTL responses are found in TKI-elicited major molecular response (MMR), when the leukemic cell load decreased, indicating efficient recovery of anti-leukemic effector responses, which are decreased at CML diagnosis (Hughes et al., 2017; Hughes and Yong, 2017). In most of CML patients on imatinib, CTL responses have been identified (Chen et al., 2008a), suggesting that leukemic-effector responses are recovered (Bocchia et al., 2006;Comoli et al., 2017).

The immunostimulatory effects of TKIs also extend to suppressor cells. The frequency of regulatory T lymphocytes (Treg) is higher in patients with CML at diagnosis than in healthy controls, which could contribute to the anergy observed at CML diagnosis. Treg numbers are reduced in TKI-treated patients (Hayashi et al., 2012). However, Treg numbers are lower in patients who control leukemia achieving deep molecular response (DMR) on imatinib therapy (Kreutzman et al., 2010; Zahran et al., 2014). Dasatinib has the potential to reduce Treg and a Treg-suppressor factor (sCTLA-4) (Nomura et al., 2019), especially in patients developing large granular lymphocytes (LGLs) lymphocytosis (Nomura et al., 2019) and NK cell differentiation, promoting immune stimulation (Najima et al., 2018). Furthermore, myeloid-derived suppressor cells (MDSCs) achieved lower levels, after imatinib (Giallongo et al., 2014) or dasatinib treatment of CML patients (Christiansson et al., 2015), with values similarly to healthy controls. However, only dasatinib-treated patients showed a reduction in the monocyte subset of MDSC (M-MDSC), which was positively associated with an MMR (Giallongo et al., 2018).

## LGL Expansion in Dasatinib-Treated Patients

Dasatinib showed the special capacity to develop LGL lymphocytosis (Mustjoki et al., 2009). The components of LGLs are mainly cytotoxic cells (CD8^+^, γδT cells, and NK cells) and are present in a half of patients receiving dasatinib (Qiu et al., 2014). Surprisingly, dasatinib mediated LGL expansion is associated with a better anti-leukemic response in both CML and Ph^+^ ALL (Mustjoki et al., 2009; Kreutzman et al., 2010; Mustjoki et al., 2013; Schiffer et al., 2016).

In dasatinib-treated CML patients, a dominant LGLs CD8^+^ TCR-Vβ^+^ expansion of either oligoclonal or polyclonal origin was recently found, reflecting multiple antigen specificities (Lissina et al., 2018). These CD8^+^ T-cell populations resembled healthy memory CD8^+^ T-cells (Lissina et al., 2018). Moreover, memory T cells are more resistant to dasatinib effects than naïve T-cells (Weichsel et al., 2008). Treatment with dasatinib might promote an increase of memory CD8^+^ T-cells, probably similar to the increased memory CD8^+^ T-cells found in primary CMV infection (Rossi et al., 2007). Further studies are therefore required to explore the mechanisms involved in the increase of CD8^+^ TCR-Vβ^+^ subpopulation that could have cytotoxic activity against CML cells in patients treated with dasatinib (Rossi et al., 2007).

In addition, a recent study identifies that LGLs are also composed of CD57^+^ cytotoxic CD4^+^ T cells with anti-leukemic response (Watanabe et al., 2018).

Regarding NK cells, dasatinib treatment of NK cells promotes cytokine expression and cytotoxic activity able to kill leukemic cell lines (Hassold et al., 2012). Hayashi et al. (2012) reported that CML patients treated with dasatinib presented an increased number of NK subpopulations such as classical NK cells (CD3^−^CD56^+^) and matured NK cells (CD56^+^CD57^+^) comparing with imatinib or nilotinib treated patients (El Missiry et al., 2016). In fact, a more mature and cytotoxic profile was found (CD57^+^CD62L^−^) in NK cells from MMR treated patients, reestablishing the NK cell functionality that was lost before any treatment (Hughes et al., 2017). Patients treated with dasatinib presented a low expression of inhibitor markers (KIR2DL5A, KIR2DL5B, and KIR2DL5), which were associated with improved molecular response at 12 months of treatment (Kreutzman et al., 2012). Whereas patients treated with imatinib had an increased expression of NK activating receptors (NKG2D and NKp30, NKp46, NKp80), a recent study has suggested that the reduced expression of NKG2A in NK cells mediated by dasatinib potentiates NK cytotoxic activity and promotes MMR in CML individuals (Chang et al., 2018).

## Dasatinib Increase Memory NK Subsets With Anti-leukemic Activity and Against CMV

LGL expansion has been often observed in dasatinib-treated patients, a phenomenon correlated with higher therapeutic responses (Kim et al., 2009). Remarkably, some authors have postulated that the development of LGLs with dasatinib is associated with prior immunity to CMV (Kreutzman et al., 2010; Kreutzman et al., 2011) but not to an increase in CMV viral load (Kreutzman et al., 2011; Ishiyama et al., 2017).

**Figure 1:**
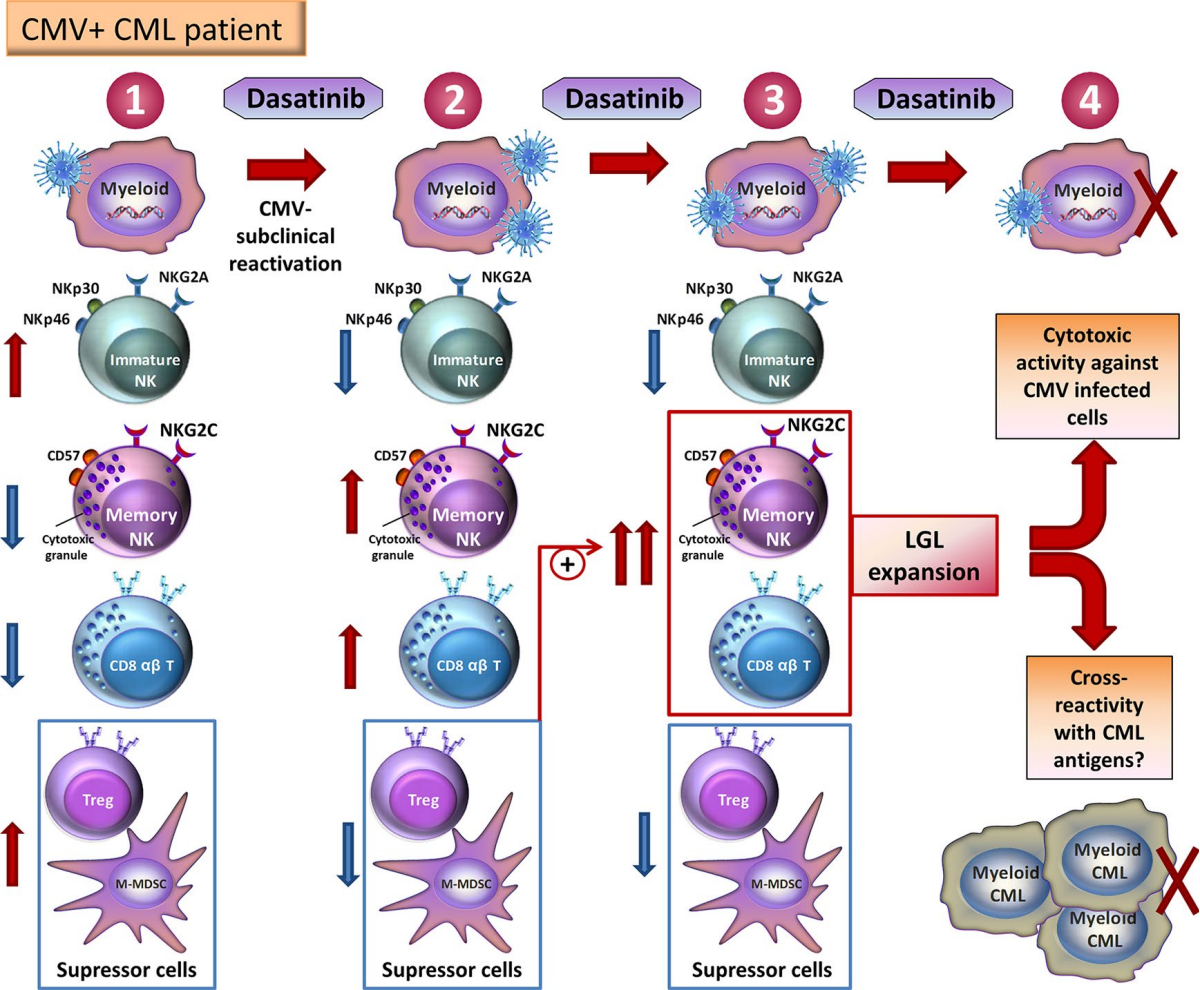
Model showing the potential mechanisms that drive NK cell and CD8^+^ T cell expansion in CMV seropositive dasatinib-treated patients with CML. (1) Before dasatinib-treatment, CMV-latent infected cells underwent a low level of CMV reactivation in CML patients. Some NK cells are immature NK cells (NKG2A^+^ NKG2C^-^NKp30^high^NKp46^high^) and a small number of CMV-associated NKG2C^+^ CD57^+^NK cells and memory CD8^+^ T-cells have been generated. However, a high amount of suppressor cells, such as Tregs and M-MDSC, is found. (2) Dasatinib *in vivo* leads to cycles of immunosuppression, which enhance subclinical reactivation of CMV and provide a replicative niche for CMV-specific memory NK cells and memory CD8^+^ T-cells. On the other hand, dasatinib treatment reduces immature NK cells load and suppressor cells amount. (3) These reduced suppressor cell load potentiates a larger expansion and effector function of CMV-specific memory NK cells and memory CD8^+^ T-cells. (4) These cells are recognized as LGLs and mediated cytotoxic activity against CMV improving disease outcomes as a consequence of CML-associated antigen cross-recognition. Adapted from Kadowaki et al. (2017).

Ishiyama et al. (2017) found that NK cells are the main component of LGLs in patients treated with dasatinib and expansion of NK cells was highly associated with being CMV-seropositive. Multiple markers on NK cells in TKI-treated Ph^+^ leukemia patients and healthy individuals were evaluated by principal component analysis (PCA) (Ishiyama et al., 2017). PCA established that NK cells from CMV^+^ dasatinib-treated patients presented a phenotype with characteristics similar to CMV-associated highly differentiated status (NKG2C^high^ NKG2A^low^ CD57^high^ LIR-1^high^ NKp30^low^ NKp46^low^). Imatinib and nilotinib treated patients and healthy individuals, that both were CMV^+^, presented a transitional profile on NK cells, and NK cells from CMV-uninfected individuals were negative for the CMV-related signature (Ishiyama et al., 2017). CMV reactivation was found in 23% of CMV^+^ dasatinib-treated patients (a positive result of PCR or elevation of CMV-IgM). Remarkably, the CMV-associated phenotype of NK cells was previously detected at diagnosis in almost all CMV^+^ patients and was further expanded after dasatinib treatment in nearly all CMV^+^ patients (Ishiyama et al., 2017). CMV^+^ patients at leukemia diagnosis had CMV replication (40%). Notably, higher grade of NK cell differentiation at diagnosis predicts both a greater expansion of CMV-adaptive NK cells and a lower leukemic cells load after dasatinib treatment in CMV^+^ patients (Rölle and Brodin, 2016; Ishiyama et al., 2017). Kadowaki et al. (2017) suggested that a low level of persistent CMV reactivation, often subclinical, triggered adaptive NK cell expansion and suggest that CMV was the first factor followed by leukemia and dasatinib as enhancing elements implicated in the NKG2C^+^ CD57^+^ cell expansion. This hypothesis is summarized in **Figure 1**.

Remarkably, the NK CMV-related signature (NKG2C^+^CD57^+^) could correspond to the human phenotype of memory NK cells, and get unique adaptive and editing properties (Rölle and Brodin, 2016). It is likely that the expansion of adaptive NK cells by dasatinib could indicate the acquisition of adaptive memory. Due to the fact that these memory NK cells are able to control leukemia in transplanted patients with CMV reactivation (Schlums et al., 2015), these NK cells could be implicated in leukemic cell control in both transplant and dasatinib-treated patients (Kadowaki et al., 2017). Therefore, the expansion of such adaptive NK cells could have long-term memory and cytotoxic effects against CML (Kadowaki et al., 2017), including after dasatinib interruption. Overall, these results suggest that dasatinib immunomodulatory effect on immunity against CMV could be used against malignancies or viral infections (Duerkop and Hooper, 2013), such as HIV infection.

## LMC Control and Therapeutic Treatment Interruption: Immunological Factors Involved in Successful Treatment-Free Remission (TFR)

A relevant aspect about treating CML is the possibility of stopping TKI treatment after reaching a DMR. In selected cases of patients with CML, treatment may be interrupted and a TFR is observed. In these patients, an immune mechanism of control by increased anti-tumor cytotoxic activity induced through chronic administration of TKIs has been suggested (Kimura, 2016), representing an alternative “curative” therapy for CML. DMR occurs in ∼20% of imatinib treated patients; nevertheless, dasatinib and nilotinib treatments allow a stronger DMR, enhancing the possibility to achieve the longest TFR (Cortes et al., 2016). Therefore, revealing the factors that could predict longer TFR might be of paramount importance (Cortes et al., 2019; Guru Murthy and Atallah, 2019). Recent stopping treatment trials have been performed to elucidate predictive factors previously found before stopping the TKI therapy, which are associated with maintained TFR (Saußele et al., 2016). Most of the stopping individuals were able to keep TFR during longer periods than a year. In fact, high levels of NK cells (Mizoguchi et al., 2013; Imagawa et al., 2015; Rea et al., 2017a), but not T cells (Imagawa et al., 2015; Ilander et al., 2017), associate with longer TFR, indicating that NK cells had a key role in maintaining CML under control.

### Imatinib Discontinuation Trials

Expression levels of NK receptors were determined in Australian CML patients prior and after TKI cessation (Hughes and Yong, 2017). NKG2D levels in NK cells were shown to be associated with CML controller patients (Hughes et al., 2016). In further EURO-SKI study, patients controlling CML expressed low levels of CD16 marker (CD16^-^) on NK cells in a cytotoxic assay using a leukemic cell line as a target (K562 cells), representing higher NK cytotoxic functionality. In fact, the amount of IFN-γ and TNF-α in NK cells (CD56^dim^CD16^−^) was associated with an effective TFR (Ilander et al., 2017).

Immunological mechanisms have been postulated to be responsible for TFR success such as IFN treatment (Ilander et al., 2014), and the increase of NK cells, especially mature NK cells (CD57^+^) and cytotoxic (CD16^+^ and CD57^+^) NK cells (Ilander et al., 2017). Actually, prior IFN therapy of patients contributes to higher control after treatment interruption, and these IFN-treated patients also presented increased NK cell counts (Burchert et al., 2015).

The only difference in immune suppressors cells observed in an imatinib trial was decreased M-MDSC at the time of TKI discontinuation in successful TFR (Hughes and Yong, 2017). It is possible that decreased M-MDSC could promote effector NK responses (Wesolowski et al., 2013), which are a necessary component for controlling leukemic cell.

### Dasatinib Discontinuation Trials

Many other TFR stopping trials are currently ongoing and evaluating discontinuation of second-generation TKIs (Rea et al., 2017b). Most of the dasatinib interruption trials such as the DADI trial (Imagawa et al., 2015; Okada et al., 2018) showed that a previous increase of NK cells (CD56^+^), LGL NK cells (CD56^+^CD57^+^), and Treg (CD25^+^CD127^low^) levels in dasatinib treatment interrupted patients was linked to a better TFR success. Recently, a case report study described that persistent memory of CD8^+^ T cells and NK cells was observed in a CML patient after more than 2 years of TFR and deep leukemic control was found after dasatinib cessation (Jo et al., 2018). In addition, a critical role of Treg inhibition by dasatinib was suggested, inducing NK cell effector differentiation and reaching DMR (Yoshida et al., 2016). Dasatinib is able to block Treg functionality (Najima et al., 2018). Therefore, probably the increased NK cell immunity and reduced immune suppressive Treg levels found in DADI individuals undergoing TFR could reduce the risk of relapse in CML patients following dasatinib discontinuation (Takaku et al., 2018).

## Dasatinib Is Able to Inhibit HIV Replication: Potential Use of Tyrosine Kinase Inhibitors in the Setting of HIV-1 Infection

Infection caused by HIV-1 is nowadays a chronic disease due to a highly efficient antiretroviral treatment (ART) that is nevertheless unable to eliminate the virus from the organism. Viral reservoirs represent the whole virus integrated in the cells and make HIV-1 infection currently incurable (Ho et al., 2013). Essential virological and immunological processes such as the activation and mass destruction of CD4^+^ T cells and the establishment of viral reservoirs are developed during early stages of infection. Unfortunately, even an effective ART started in early stages cannot fully eliminate viral reservoirs (Buzon et al., 2014;Laanani et al., 2015; Bruner et al., 2016). Then, new strategies are needed to reduce viral reservoirs or to directly prevent their establishment by additional mechanisms including immunotherapy and new immunomodulatory compounds in order to cure the infection (Coiras et al., 2017).

The family of TKIs is directed against the activation of TK. Some of these kinases are essential for the activation of CD4^+^ T cells, the main HIV-1 target. Consequently, we discuss the possibility of using TKIs in combination with the ART in HIV-1 infection especially during the acute/recent phase (Coiras et al., 2017).

As far as we know, only a French group (Pogliaghi et al., 2014), our Spanish group (Bermejo et al., 2016; Bermejo et al., 2018), and recently a North American group (Szaniawski et al., 2018) have developed research related to dasatinib as a potential HIV therapy. We have shown that dasatinib is able to inhibit the phosphorylation of SAMHD1 into CD4^+^ T cells. SAMHD1 is an innate antiviral restriction cell factor that acts by decreasing the levels of intracellular dNTPs below the level required for proper viral replication, and whose phosphorylation inactivated this restriction. Therefore, dasatinib inhibits HIV-1 replication preserving the HIV-1 antiviral activity of this innate factor in CD4^+^ T cells (Bermejo et al., 2016; Bermejo et al., 2018) and also in human macrophages (Szaniawski et al., 2018). Additionally, their antimitotic properties can reduce the clonal expansion of infected cells carrying HIV-1 provirus, decreasing the permanent filling of viral reservoirs (Coiras et al., 2017). Interestingly, new data from the Spanish group demonstrated that dasatinib is safe and inhibits HIV-1 infection in an *in vivo* humanized mice model (Salgado et al., 2019). Moreover, dasatinib could reduce inflammation and senescence in *in vivo* and *ex vivo* models, which are two major problems of ART-treated HIV infected patients (Xu et al., 2018; Short et al., 2019). Nevertheless, there are some safety concerns using dasatinib in HIV-1 patients: a) possible drug interactions with ART or b) some adverse effects such as infectious complications. Actually, dasatinib is metabolized by cytochrome P450 3A4 (CYP3A4) and ART containing CYP3A4 inhibitors (cobicistat or ritonavir) are not recommended (Ambrosioni et al., 2017). However, other ART regiments including integrase inhibitors are appropriate due to the lack of interaction with dasatinib (Bermejo et al., 2018). Dasatinib side effects found in some CML studies could be avoided with low doses and shortened dasatinib treatments in HIV patients. In fact, effective doses for HIV inhibition are lower than the one administered in CML. Moreover, selecting a cohort of HIV patients with high CD4 counts could reduce the risk of infections (Bermejo et al., 2018). Furthermore, little evidence of the use of dasatinib was found in association with ART therapy in HIV patients as a few ART treated HIV-1 patients also have CML. However, some HIV-infected patients developed CML and then started dasatinib treatment showing good dasatinib adherence and an excellent CML control (Patel et al., 2012; Campillo-Recio et al., 2014).

Additionally, the immunomodulatory role of dasatinib is also supported by the fact that peripheral blood lymphocytes obtained from CML patients dasatinib treated for at least 6 months with dasatinib appear to be resistant to HIV-1 infection (Bermejo et al., 2016; Bermejo et al., 2018). Because resistance to infection by viruses such as CMV and HIV share immune response pathways similar to anti-tumor activity, it would be interesting to determine how TKIs might be inducing cytotoxic and immunoregulatory responses that simultaneously affect HIV-1 infection and control of CML.

Interestingly, some studies have recently demonstrated that adaptive NKG2C^+^ CD57^+^ NK cells have found to be associated with control of HIV-1 in early infected patients (Gondois-Rey et al., 2017; Flórez-Álvarez et al., 2018; Peppa et al., 2018), suggesting that dasatinib could promote control of HIV viremia by expanding those memory NKG2C^+^ CD57^+^ NK cells against HIV-infected cells in CMV^+^ patients [most than 83% of HIV patients are co-infected with CMV (Lichtner et al., 2015)]. Moreover, NKG2C is an NK receptor that binds the HLA-E. HLA-E antigen presentation has been implicated in the induction of a very potent cytotoxic response against HIV mediated by CMV vectors (Hansen et al., 2011: Hansen et al., 2016). Thus, dasatinib and CMV would potentiate the emergence of this potent NK response mediated by the NKG2C receptor that might eliminate CMV-infected cells, leukemic-cells, and, likely, HIV-infected cells. Noteworthy, low NKG2A expression in NK cells was correlated with HIV control (low viral load) (Ramsuran et al., 2018). Therefore, therapeutic reduction of NKG2A expression in NK cells mediated by dasatinib (Chang et al., 2018) could promote HIV control blocking HLA-E:NKG2A interaction and yielding benefit against HIV infection.

Finally, therapeutic strategies aimed at inhibiting MDSC, such as decreasing M-MDSC using dasatinib (Giallongo et al., 2018), might not only further enhance anti-leukemic cytotoxicity (Gabrilovich and Nagaraj, 2009), but also potentiate an effective immune response against viral infections such as HIV infection.

**Figure 2:**
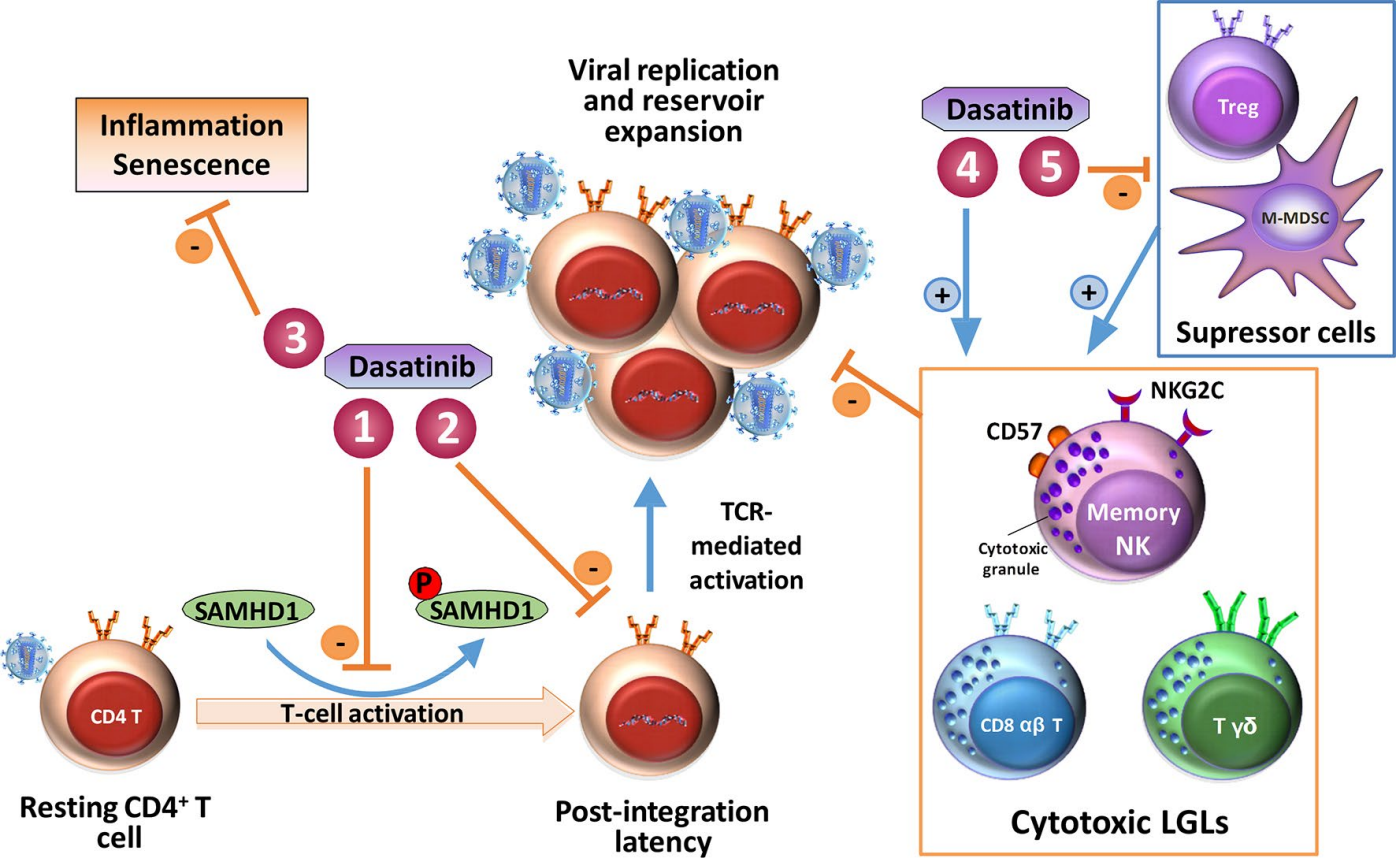
Schematic representation of dasatinib mediated mechanisms that could interfere with HIV-1 infection. (1) Inhibition of SAMHD1 phosphorylation, maintaining its intrinsic antiviral activity; (2) inhibition of mass activation, establishment of viral reservoirs, depletion of CD4^+^ T cells, and reduction of infected cells that carry the pro-viral DNA of HIV-1; (3) inhibition of inflammation and reduction in senescence; (4) potentiation of cytotoxic activity mediated by memory NK cells and T lymphocytes against HIV; (5) increase of immune effector cells such as cytotoxic LGLs by reducing levels of Tregs and M-MDSC, which are suppressor cells. Adapted from Coiras et al. (2017).

Taken together, dasatinib could act against HIV-1 by various mechanisms (showed in **Figure 2**):

1. Inhibition of SAMHD1 phosphorylation, maintaining its intrinsic antiviral activity;
2. The inhibition of mass activation and, consequently, of the establishment of viral reservoirs and the depletion of CD4^+^ T cells and reduction of infected cells that carry the pro-viral DNA of HIV-1;
3. Administration of dasatinib to treat HIV-1 infection could reduce activation of T cells decreasing chronic inflammation and providing a reduction in senescence;
4. The potentiation of cytotoxic activity mediated by memory NK cells and T lymphocytes against HIV (similar to that observed against leukemic cells in CML-patients treated with dasatinib) (Ambrosioni et al., 2017);
5. Increasing immune effector functions by reducing levels of Tregs (Najima et al., 2018) and M-MDSC.

## Concluding Remarks and Perspectives

In conclusion, the elucidation of the immunomodulatory mechanisms that may be associated with TFR after suspension of TKIs is essential to establish a series of more precise treatment interruption parameters and determine the immunomodulatory capacity of these drugs, not only in the field of CML, but possibly of other tumors favored by tyrosine kinases as well as in CMV and HIV-1 infection.

Dasatinib treatment in CML induces populations of NK, NK-LGL, and T-LGLs that are associated with better prognosis of CML, a possible indefinite TFR and good cellular response against CMV. Our studies described that TKIs prevent the replication of HIV-1. A future goal is to explore whether TKIs such dasatinib could safely prevent HIV-1 replication in HIV patients not only directly inhibiting SAMHD1-phosphorilation but also indirectly promoting the expansion of a highly effective HIV-specific immune response mediated by NK (NKG2C^+^CD57^+^) cells and polyclonal CD8+ TCR-Vβ^+^ cells, likely better expanded in CMV^+^ patients. Consequently, the use of dasatinib in combination with ART or new combined immunotherapies could protect CD4^+^ T cells and macrophages from infection and activate a powerful cytotoxic response that could promote the elimination of the HIV-1 reservoir and reduce HIV associated inflammation and senescence.

## Author Contributions

NC and MP conceived the review and wrote the manuscript. Both authors approved the final manuscript.

## Funding

This study was partially supported by grants: FIS PI15/00480, AC16/00051, and FIS PI18/00699 from ISCIII co-financed by Fondo Europeo de Desarrollo Regional (FEDER); RETIC-RIS RD16/0025/0002; and Department of Health within the framework of the HIV Vaccine Development in Catalonia (HIVACAT) program.

## Conflict of Interest

The authors declare that the research was conducted in the absence of any commercial or financial relationships that could be construed as a potential conflict of interest.
